# Cancellous compression bone grafting using headless screw as a strut in scaphoid nonunion by a single volar approach

**DOI:** 10.1007/s00590-023-03485-2

**Published:** 2023-02-20

**Authors:** Anil K. Bhat, Sourab Shetty, Ashwath M. Acharya

**Affiliations:** Department of Orthopaedics, Kasturba Medical College-Manipal, Manipal Academy of Higher Education (MAHE), Manipal, Udupi, Karnataka 576104 India

**Keywords:** Scaphoid nonunion, Compression grafting, Pure cancellous grafting, Scaphoid volar approach, Scaphoid screw fixation

## Abstract

**Purpose:**

An array of fixation and grafting techniques for scaphoid nonunion have been described over time, achieving varied results pertaining to union and scaphoid alignment. The aim of this study was to check for union rates and correction of scaphoid parameters achieved by our technique of using screw as a strut and cancellous compression grafting harvested from the distal radius by a single volar approach.

**Methods:**

Retrospective analysis of all patients operated by the said technique was done from 2013 to 2019. Thirty-eight patients including 36 males and two females in the age range of 20–56 years were analyzed. Union rates, change in scaphoid alignment parameters and graft site characteristics were documented.

**Results:**

Of the patients analyzed, 5/38 were nonunions of proximal pole, 19/38 of waist and 14/38 were of distal pole which included nonunions of cystic type or with humpback deformity. Patients were followed up for an average of 22.2 months and union was achieved in all cases with a mean period of 15.7 ± 3.7 weeks. There was significant improvement in the scaphoid alignment postoperatively. One case of implant migration and one case of scaphoid nonunion advanced collapse were noted. The mean duration of donor site healing was noted to be 16.9 ± 2.5 weeks except two outliers which took longer time. There were no cases of donor site fracture or other complications.

**Conclusion:**

Union rates, correction of scaphoid parameters and minimal complications justifies this technique as a novel one in the management of scaphoid nonunion at all levels, with minimal donor site morbidity and attained by the single volar approach.

## Introduction

The scaphoid forms an important biomechanical link between carpal rows and is critical in wrist movements and stability [1]. Nearly 80% of the surface of scaphoid, including the entire proximal pole, is covered by articular cartilage limiting bony contact for fracture healing [2, 3]. Moreover, the medullary canal has minimal vascularity and the humpback deformity resulting from displacing forces, make nonunion one of the most common complications [2–4]. The incidence of nonunion in scaphoid fractures is 5–12% and with a displacement of greater than one mm at the fracture site, it may be as high as 55% with complications such as carpal malalignment, scaphoid nonunion advanced collapse (SNAC) and progressive radiocarpal and midcarpal arthritis in the long run [5–7].

Surgical treatment is indicated in scaphoid nonunion to re-establish the scaphoid length, correct the flexion posture and achieve bony union by replacing the fibrous tissue with an osteoinductive and osteoconductive matrix and fixing with a stable construct [2, 8]. Scaphoid alignment, achieved by a lateral intrascaphoid angle of less than 35°, also plays a key role in regaining the function and decreasing the probability of arthritis [9].

The evolving and challenging surgical management of scaphoid nonunion has encompassed various fixation and grafting techniques. The screw functions as an internal strut to achieve and maintain alignment and length, when it is placed before the placement of graft, maintaining the fact that the fracture segments are large enough to support fixation [9, 10]. Numerous studies have also been done on the implications of various vascularized and non-vascularized bone grafting [11]. Pure cancellous grafts have been found to have equally good osteoconductive and osteoinductive properties when adequate fixation of the nonunion site is done [12].

We have often been impressed by some amount of dorsal contact of the cartilage shell whenever we have operated a case of chronic scaphoid fracture nonunion, including the ones with a humpback deformity. With a volar approach, overhang of the distal segment of the fracture can be corrected and fixed with a single screw to achieve the length and alignment and then augmentation can be done with well compressed cancellous graft, which further provides structural support thereby precluding the need for a corticocancellous graft from a distant site. We therefore describe a technique of treatment in nonunions of scaphoid in the form of cyst formation, humpback deformity or minimal dissociation regardless of the location of nonunion, using a single headless screw augmented with compressed cancellous autograft from the distal radius via a single volar approach. The aim of the study was to assess fracture union rates achieved with this technique and correction of alignment and length achieved, confirmed by radiological parameters. The graft donor site characteristics were also observed during follow-up.

## Methods

We retrospectively reviewed patient records of all cases of nonunions of scaphoid managed by the said technique between 2013 and 2019. Patient baseline demographic data including age, gender, side and duration since injury along with radiological and operative data were reviewed from our medical records department and our internal radiological database, the Picture Archive and Communication System (PACS). The study was started after appropriate permission from the Institutional Ethics Committee. (IEC 464/2020).

Preoperatively standardized plain radiographs were taken, which included the wrist posteroanterior (PA) view, lateral view and Billiard view. Computerized Tomography (CT) scan with 1 mm slices along the scaphoid long axis and Magnetic Resonance Imaging (MRI) were not undertaken routinely except in cases with suspicious avascular necrosis (AVN) of the proximal segment, radiocarpal or midcarpal arthritis on plain radiographs and to look for displacement and comminution. A persistent fracture gap with sclerotic and/or cystic changes at the site of fracture along with bone resorption, as seen on simple radiography at even three months after the trauma was defined as nonunion [13].

**Fig. 1:**
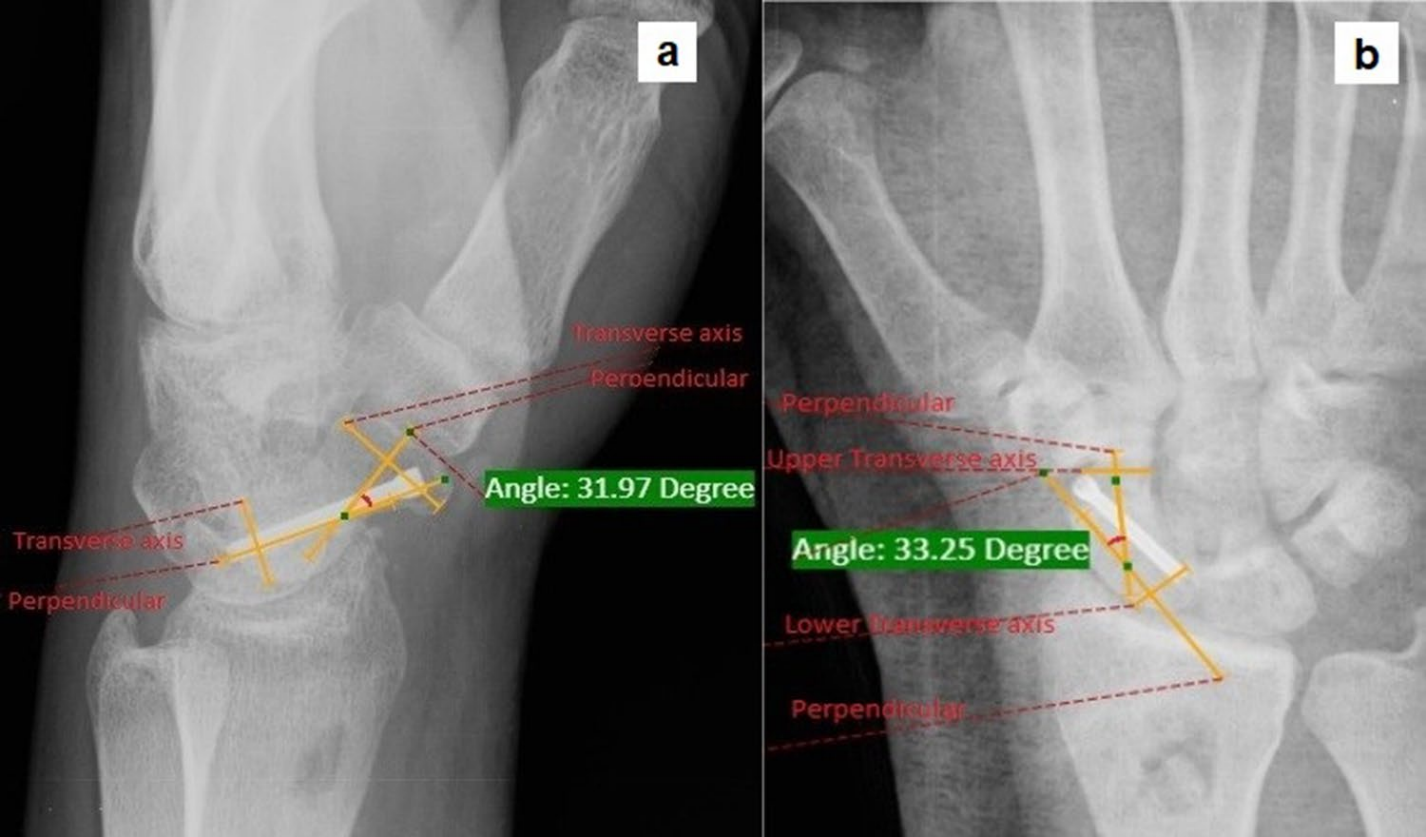
Intrascaphoid angle (ISA) **a** Lateral view **b** Posteroanterior view

**Fig. 2:**
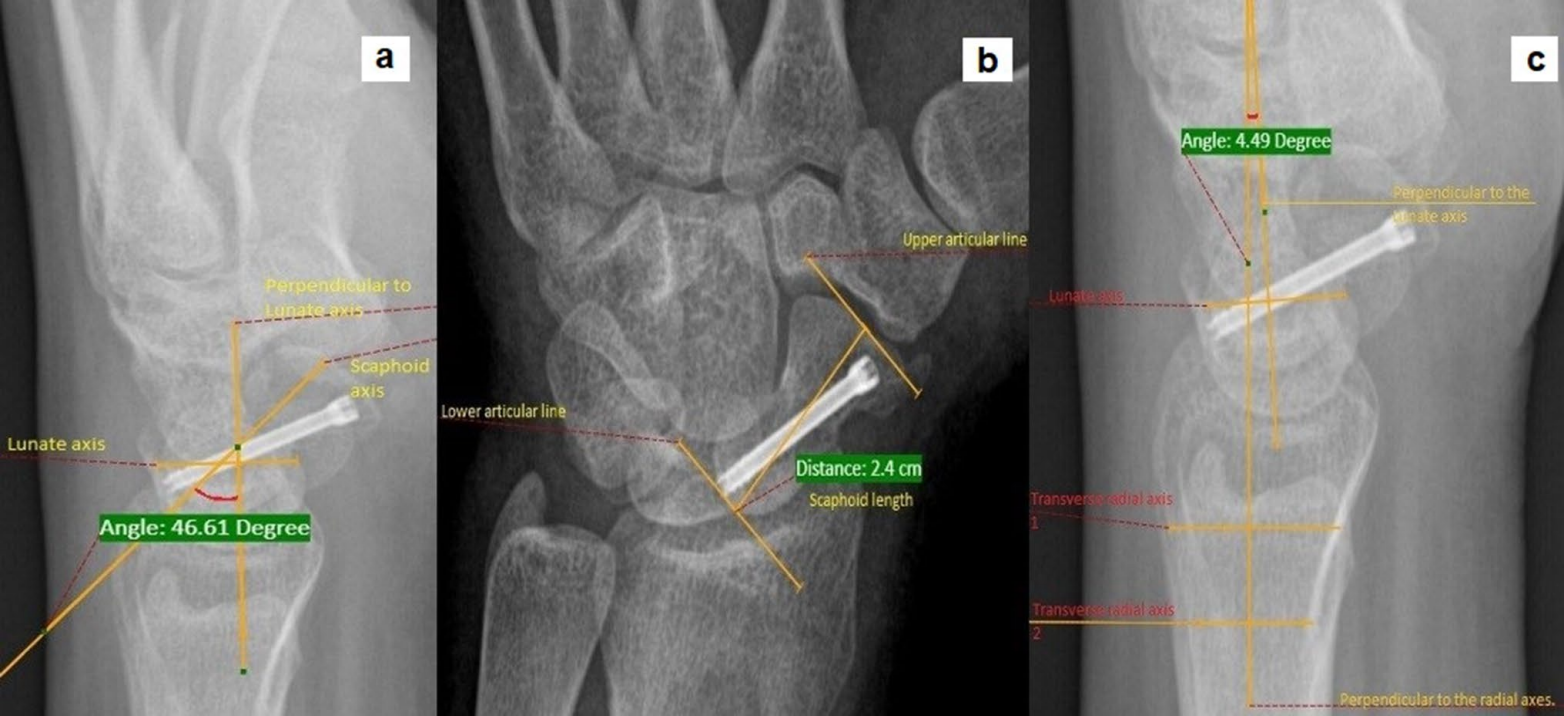
Scaphoid parameters **a** Scapholunate angle (SLA) **b** Scaphoid length in Billiard's view **c** Radio lunate angle (RLA)

For radiological evaluation, Intrascaphoid Angle (ISA)—both in lateral and PA radiograph (Fig. 1), Scapholunate Angle (SLA), scaphoid length (SL) and Radio Lunate Angle (RLA) were measured preoperatively, postoperatively and in the last follow-up (Fig. 2). Bony union was defined as bridging bone noticed on orthogonal views showing consolidation of the nonunion gap as seen on serial follow-up radiographs [14]. Lateral radiographs were used for measuring SLA and RLA and Billiard's view was used for scaphoid length on the PACS system. Though the range of normal values of RLA, SLA [15, 16] and ISA [9] are described in previous literature, we considered the contralateral normal wrist of the individual patient as the reference for correction of parameters. In addition, we also looked for complications such as proximal fragment AVN, screw displacement and SNAC wrist in the serial follow-up radiographs. The size of the graft donor site, the distance of it from the articular surface and the time taken for the donor site to heal were also documented.

### Surgical technique

**Fig. 3:**
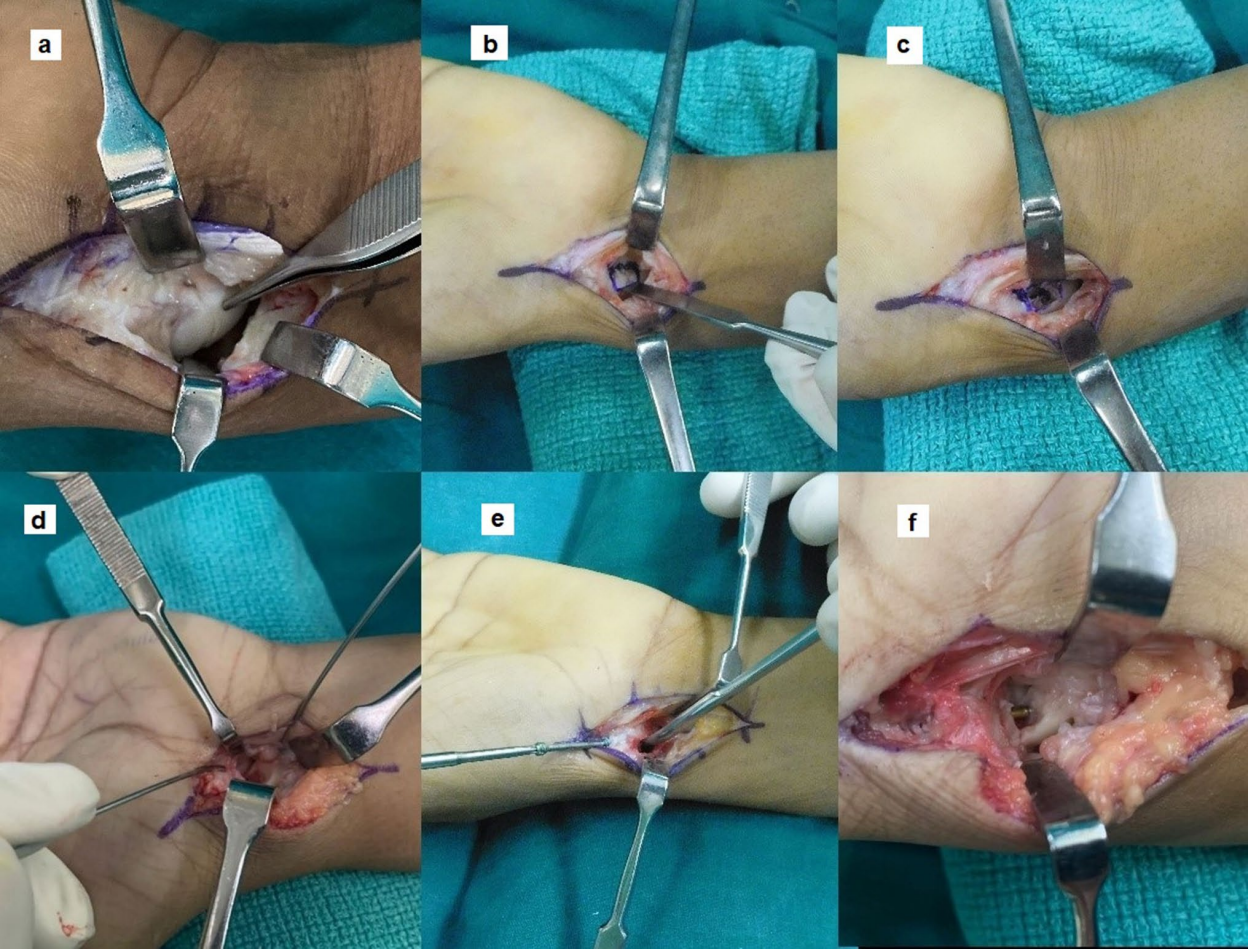
Single volar approach with a hockey stick-shaped incision to expose both the scaphoid and distal radius **a** Fracture line over the volar shell of scaphoid **b** Window over the volar shell **c** Opening the nonunion site **d** Joysticks placed and nonunion site debrided **e** Headless screw passed while maintaining the alignment and gap **f** Screw functioning as a strut

The patient was positioned supine with the arm over an arm table. All the cases in our study were operated by a single trained hand speciality surgeon in our Level 3 trauma center. Our technique utilized a volar approach, with a hockey stick-shaped incision positioned over the scaphoid long axis and extending proximally along the radial aspect of the flexor carpi radialis tendon. The non-articular, volar aspect of the scaphoid was exposed until the proximal pole by a longitudinal capsulotomy until the distal radius volar margin. The typical observation here would be either a normal looking scaphoid with an intact volar shell but with a fracture line or a discontinuous volar shell with a flexed pronated overhanging distal pole (Fig. 3). Two 1 mm K-wires were then placed as joysticks in the distal and proximal segments and the nonunion site was opened by extending and supinating the distal segment usually exposing an intact posterior shell in this gap (Fig. 3). Hump back deformity when gently pried open also would lead to contact at the posterior cortex.

Subsequently, debridement of the nonunion site was done using nibblers and serial size of curettes until fresh punctate bleeding was noted. A low speed burr was used at times to create fresh bleeding surface. The two segments were then held in alignment using the joysticks and a guide wire was passed from the distal pole to the proximal under image guidance. The correction of the alignment was checked both in PA and lateral views and the relative neutral alignment of lunate was checked to confirm the correction. The central placement of the guidewire was also noted. In proximal pole fractures, the entry was taken slightly volar and kept perpendicular to the fracture site to get better purchase of the small proximal fragment. The length was measured and a 2.4 mm headless screw was then passed by placing a bone lever in between to avoid compression of the gap using the screw as a positional screw. The joysticks were used to avoid compression and rotation (Fig. 3).

**Fig. 4:**
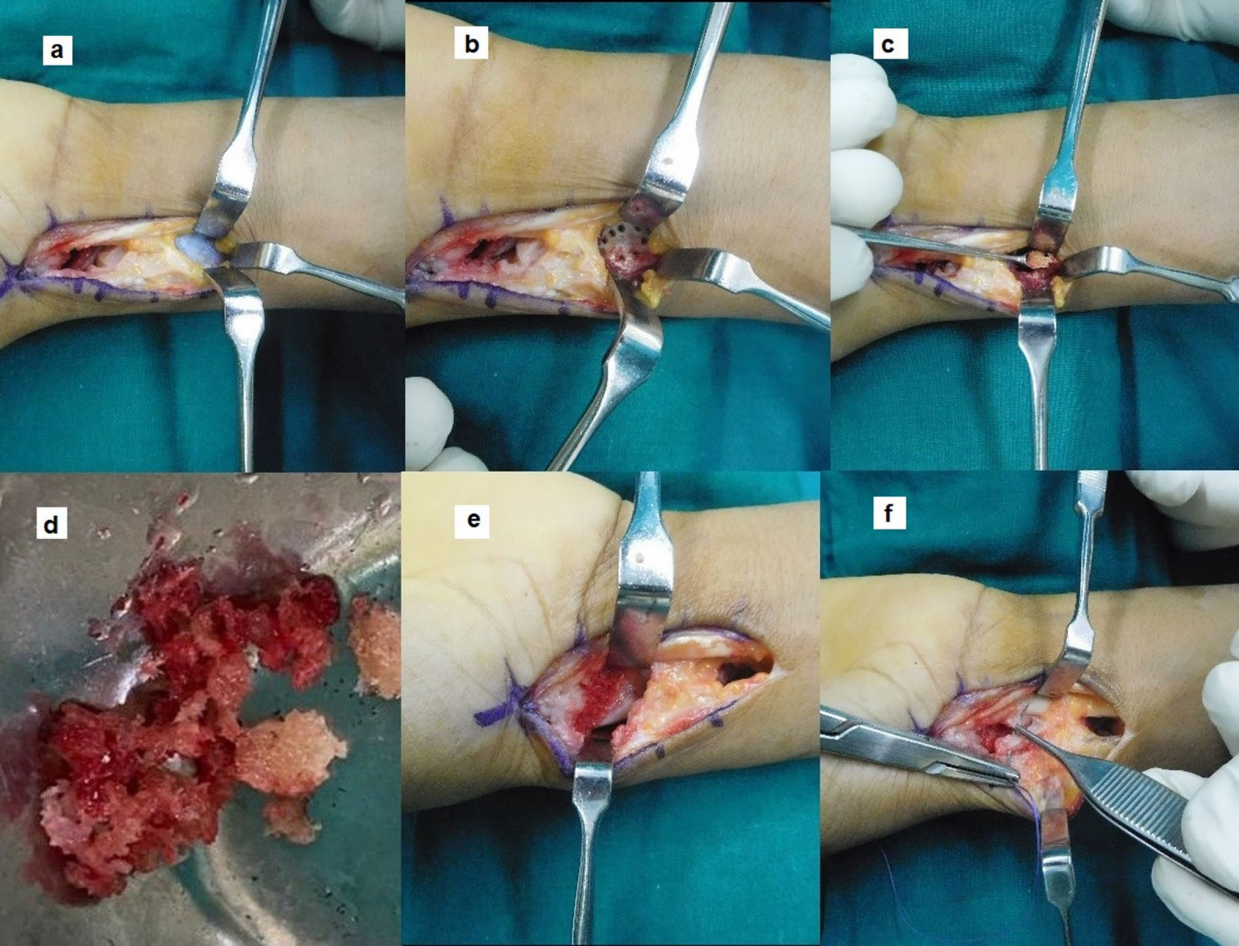
Harvesting the graft **a** Pronator Quadratus exposed through the same incision **b** Drill holes made over the distal radial cortex **c** Cortical window raised and graft harvested **d** Copious amount of morselized cancellous graft harvested **e** Graft filled and compressed from dorsal to volar around the screw into the nonunion site f Capsule closed

The proximal end of the incision over the radius was further exposed by medially retracting the flexor carpi radialis and elevating the Pronator quadratus. Drill holes were made using a 2 mm drill bit on the volar cortex of radius and a cortical window was created. Cancellous bone was harvested using curettes (Fig. 4).

Now with the screw acting as an internal strut at the site of nonunion, the cancellous graft harvested was packed tightly into the nonunion gap starting dorsally behind either side of the screw till the volar side (Fig. 4). The graft was impacted using small sized punches in increasing diameter incorporating maximum amount of graft. This further aids in nullifying the axial compressive forces acting at the fracture gap and mechanically strengthens the construct. The incision was then closed in layers and the hand was immobilized in a below elbow thumb spica slab. The approximate surgical time was around 45 min.

After the first wound inspection and subsidence of postop edema, the patient was put on a below elbow thumb spica cast for 6 weeks allowing mobilization of other fingers. Following this, a thumb spica orthosis was given for 2 weeks allowing intermittent mobilization of thumb. Patients were seen at an interval of 4 weeks until union of fracture was confirmed and every 3 months thereafter to look for complications. Restricted lifting of weights and gripping activities were advised till fracture union was confirmed.

### Statistical analysis

The demographic descriptive statistics were reported as mean ± standard deviation for continuous variables and as a percentage for categorical variables. Paired T test was used to analyze pre and postoperative radiological parameters. Pearson's correlation was used to analyze the effect of the distance of the donor site from the articular surface on the fracture healing and to look for any relation between the size of the donor site and the time taken by the donor site to heal. The results were considered statistically significant with a *p-value* < 0.001. Statistical analysis was done using *SPSS software v 20.0 IBM corporation. (SPSS Inc., Chicago, Illinois)*.

## Results

A total of 38 patients operated by the technique between 2013 and 2019 were analyzed. The study cohort included 36 males (95%) and two females (5%) with a mean age of 28.7 ± 7.9 years (Range: 20–56 years). 17/38 (45%) patients had an injury on the right hand. The dominant hand was involved in 23/38 patients (61%). Patients presented at a mean period of 18.7 ± 4.9 weeks (Range: 12–32 weeks) following the injury during which some patients had undergone prior management with cast.

**Fig. 5:**
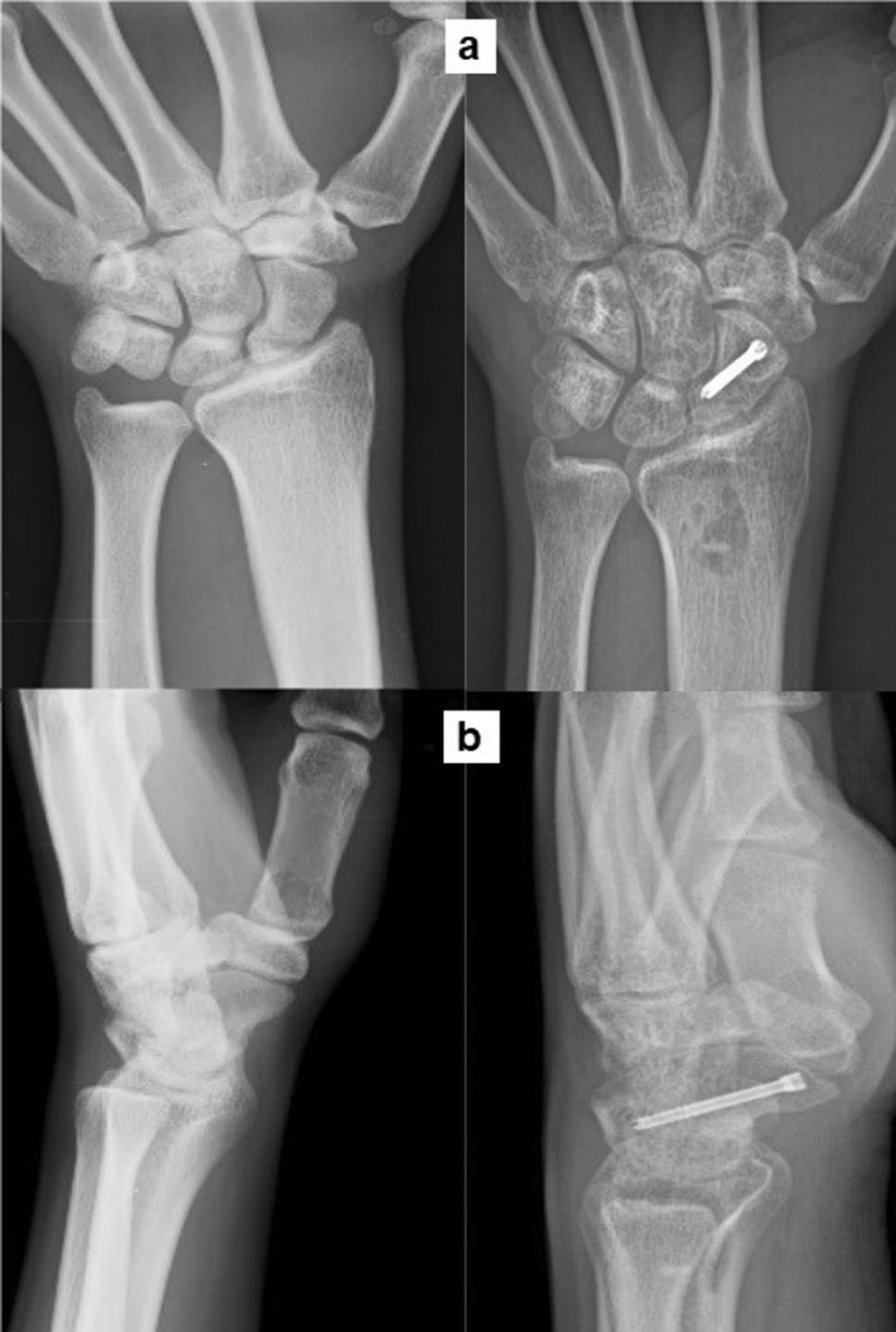
Proximal pole nonunion managed by the technique, showing union 4 months post-surgery **a** Posteroanterior view **b** Lateral view

**Fig. 6:**
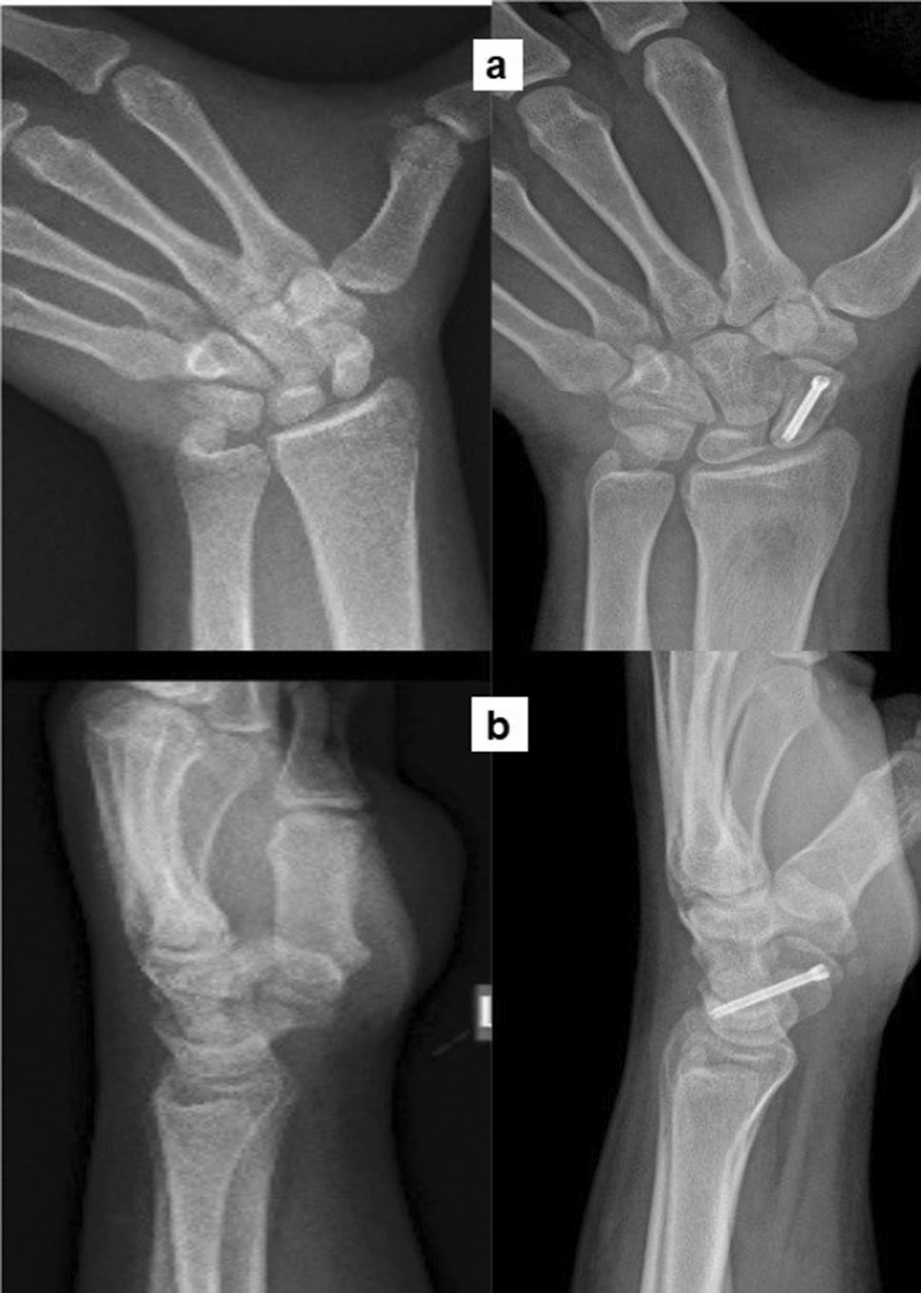
Scaphoid waist fracture nonunion showing union 4 months post-surgery **a** Posteroanterior view **b** Lateral view

**Fig. 7:**
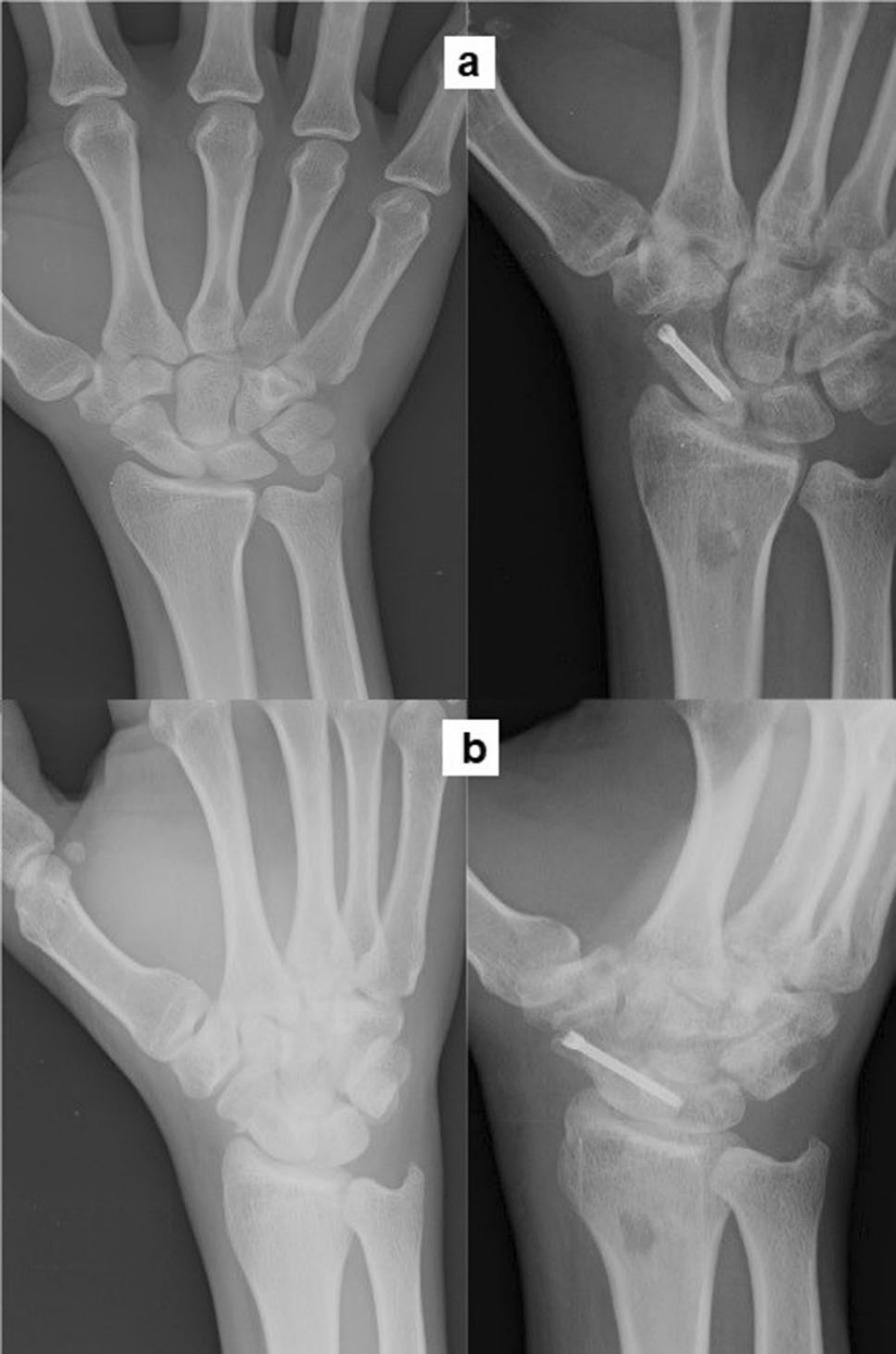
Distal pole nonunion preoperatively and 3 months postoperatively **a** Posteroanterior view **b** Oblique view

Of the 38 patients who were treated, five (13%) had proximal pole nonunion, 19 (50%) had nonunion of the waist and 14 (37%) had distal pole nonunion. 21/38 (55%) patients had pure cystic kind of nonunion, 14/38 (37%) patients had a humpback deformity and 3/38 (8%) patients had minimal dissociation at nonunion site, which were all confirmed intraoperatively and documented in the surgical notes. The patients were followed up for an average period of 22.2 months (Range: 12–48 months) and there was no loss in follow-up of any patient. Union was achieved in all cases with a mean period of 15.7 ± 3.7 weeks (Range: 10–30 weeks) (Fig. 5, 6, 7).

Significant improvement was noted in the mean ISA, SLA, RLA and SL (*p value* < 0.001) in the postoperative radiographs compared to the preoperative ones (Table 1). The patients were kept on periodic follow-up and radiographs revealed neither a deterioration in the alignment of scaphoid nor any hardware migration at final follow-up except for one case of distal pole nonunion, which showed lysis and change in the screw position at one year. However, it had united at 4 months with no changes in the radiological parameters in the final follow-up. Hence, implant removal was done and no signs of infection was noted.

**Table 1:** Analysis of radiological parameters

Parameter	N	Mean ± SD*	Mean difference ± SD*	t	*p value*
Lateral Intrascaphoid Angle—Preop	38	45.58 ± 5.74	14.65 ± 5.44	16.59	< 0.001
Lateral Intrascaphoid Angle—Postop	38	30.93 ± 2.06			
Posteroanterior Intrascaphoid Angle—Preop	38	34.01 ± 2.13	3.77 ± 1.86	12.51	< 0.001
Posteroanterior Intrascaphoid Angle—Postop	38	30.24 ± 1.22			
Radiolunate Angle—Preop	38	10.57 ± 2.65	4.35 ± 2.07	12.98	< 0.001
Radiolunate Angle—Postop	38	6.21 ± 1.23			
Scapholunate Angle—Preop	38	61.01 ± 5.26	13.88 ± 5.86	14.61	< 0.001
Scapholunate Angle—Postop	38	47.13 ± 3.04			
Scaphoid Length—Preop	38	19.72 ± 0.59	2.72 ± 0.67	25.00	< 0.001
Scaphoid Length—Postop	38	22.44 ± 0.63			

^*^—Standard Deviation

All patients showed features of healing, as documented by bony trabeculations. One patient with proximal pole nonunion had prominence of the screw at the entry point from the initial radiographs. However, it was non-articular and the patient had no complaints of pain clinically or arthrosis in the follow-up radiographs of 2.5 years. Grade 1 SNAC was noted in one case of waist nonunion at 2 years follow-up. No features of AVN were noted in any case for the period of follow-up.

The mean distance of the graft donor site from the articular surface was noted to be 9.04 ± 0.85 mm. On an average, the diameter of the donor site was 8.06 ± 0.82 mm in the longitudinal plane and 7.69 ± 0.74 mm in the transverse plane. The mean duration required for the donor site to fill up was 19.5 ± 11.5 weeks (Range: 12–72 weeks) where two cases showed a small gap to be filled up at 60 and 72 weeks follow-up also. Excluding the outliers, the mean duration of donor site healing was 16.9 ± 2.5 weeks (Range: 12–22 weeks). There was no case of fracture of the donor site and it did not hinder in the rehabilitation protocol. Neither the size of the donor site affected its healing time significantly nor did the distance of the donor site from the articular surface have any significant correlation with the nonunion healing time (*p* value > 0.001) (Table 2).

**Table 2:** Correlations checked of graft donor site with size and nonunion site healing time

Parameters being correlated	N	Correlation(r)	*p value*
Union time in weeks and Distance of graft site in millimeter	38	0.004	0.982
Vertical length of graft donor site in millimeter and Duration for graft site filling in weeks	38	0.148	0.374
Transverse length in millimeter and Duration for graft site filling in weeks	38	0.202	0.22

## Discussion

The natural history of scaphoid nonunion, if not treated, is late morbidity from pain, weakness of grip and decrease in the range of motion of the wrist along with complications such as SNAC and capitate proximal migration collapse [17, 18]. Hence, though daunting, achievement of union and alignment along with regaining the length and correcting the flexion posture in scaphoid nonunion is mandatory. There are a number of strategies described for the treatment of scaphoid nonunion based on various parameters [19] but maintaining blood supply, debridement of nonunion site, achieving correct alignment, bone grafting and stable internal stabilization are critical requirements [13].

Herbert and Fischer established headless screws to be a reliable method of fixation for scaphoid nonunion in 1984 [20]. Two 1.5 mm screws were initially claimed to have higher mechanical characteristics to a single 2.2 or 3 mm screw but now has been proven to be the same [10]. Further, other modes of fixation such as volar locking plates have been used. A recent series using volar locking plates has reported union in 13 of 15 patients (87%) at a mean period of 5 months. However, hardware complications were noted in four patients like plate breakage and screw backout while plate impingement against the radial styloid was noted in six patients [21]. Further, a biomechanical study by Beutel et al. [10] has also demonstrated that if there is some native cortical contact in the scaphoid, then the ability to withstand loads is the same in volar locking plates and headless compression screws. In our study, we have noticed most cases to have a dorsal contact of the cartilage shell and have achieved 100% union in a mean period of 4 months with lesser complications. Similarly, a study by Cohen et al. [22] reported 12 patients who achieved 100% union using only cancellous grafting and screw fixation. However, proximal pole fractures were not included in the study unlike our study. Besides, anterior trapezium resection was done for screw placement and a separate dorsal incision was given for graft harvest in the study [22]. A recent study by Tavakolian et al*.* [23] achieved 100% union with volar plate fixation and pure cancellous autograft and provided tips to appropriately use volar plates without complications. The study also highlighted the compression caused by using headless screws in scaphoid nonunions leading to malunion. The screw used in our study also has a variable pitch, which ideally provides compression but was used in view of it being a headless screw. Therefore, it was ensured that there was no loss of reduction during screw placement by holding the fracture ends apart using the joysticks and by placing a bone lever in between.

When the purchase of the screw is adequate at the distal and proximal segments, it functions as a strut until the cancellous graft is packed in tightly, which further aids in providing structural integrity and prevents the construct and thereby the gap from collapsing thereby nullifying the need for a corticocancellous interposition graft. A recent study also highlighted that the volume of the proximal pole did not correlate with increased risk of ongoing nonunion [24]. Reduction in fracture, as well as intrascaphoid angles in our study were consistent with other studies which used corticocancellous graft, measured using similar imaging techniques [25]. Also, we believe that pure cancellous graft incorporates faster than corticocancellous graft which can be attributed as a cause for the consistent union rate in our technique [12]. Furthermore, the scaphoid gap can be filled in more precisely and compactly by morselized cancellous graft than corticocancellous graft as also claimed in a recent study by Christodoulou et al.[26]. There is also no prominent difference in union rates between grafts harvested from the iliac crest or distal radius besides avoiding donor site morbidity at a distant site and pain related to iliac crest [27].

Vascularized bone grafts have gained a significant role to play in scaphoid nonunions, especially the proximal pole fractures. With modern fixation techniques, the ideology of cancellous bone grafting having no role in proximal pole nonunions may no longer hold true, as vascular inefficiency might not be the only factor affecting its union or leading to AVN [28, 29]. With a follow-up of 10 years, 100% union was reported in most fractures of scaphoid nonunion using cancellous bone graft by Finsen, Hofstad and Haugan [30]. Ramamurthy et al. [31] presented the results of 126 nonunions with 40 proximal pole fractures treated with non-vascularized bone grafting and showed a union rate of 80%. Our technique also shows a 100% union rate with pure cancellous compressed grafting, at all locations of nonunion and precludes the need of a vascularized graft and thereby the expertise and additional operative time associated with it (Table 3).

**Table 3:** Comparison pertaining to benefits and limitations of techniques available in current literature

Techniques	Advantages	Limitations
*Implant*		
a) K-wires (2 or 3) [31–34]	Can be removed at 6–8 weeks; More space for graft placement; Lesser space required for entry	Minimum 2 wires are required to provide rotational stability and to maintain length; Less stable construct than screw or plate; Pin tract infections and entry site irritation; Possibility of backout
b) Regular Screws (1 or 2) [20, 21, 31, 35]	Construct as stable as a plate if some cortical contact present; Lesser soft tissue erasure than a plate	Regular screws cause more cartilage damage and adequate compression might not be achieved if head stays prominent; More spacing out is required to accommodate the heads; Using two screws decreases the space for graft
c) Volar plate [21, 36, 37]	More stable construct; Can be used in unstable and grossly displaced fractures	Plate breakage; Screw backout; Impingement; More exposure for plate placement; Implant removal required most often
d) Single headless compression screw (Current study)	Stable construct along with the compressed graft and intact posterior shell; Single screw provides more space for graft; Even small proximal pole segments held well with single screw	Prominent screw head if not buried might cause scaphotrapezial arthritis; The results not verified in displaced fractures
*Graft*		
a) Iliac corticocancellous graft [20, 35]	Provides structural stability to prevent volar collapse	Must be shaped adequately to fit rightly into the gap; Distant donor site morbidity
b) Iliac cancellous graft [31–33, 35]	Incorporates earlier than corticocancellous grafts; Can be filled in more precisely and compactly; With stable fixation gives similar results to vascularized grafts in proximal pole fractures	Distant donor site morbidity
c) Vascularized graft [8, 34–36]	Plays an important role in nonunions with features of AVN and in proximal pole fractures	Surgical expertise required; Increases operative time
d) Compressed cancellous graft from distal radius (current study)	Gives similar results as iliac cancellous grafts; Compact filling acts as structural support along with the screw; With stable fixation gives similar results to vascularized grafts in proximal pole fractures; No distant donor site morbidity and can be done under regional anesthesia	Care of donor site till it heals (approximately 4 months)
*Approach*		
a) Dorsal [34, 36]	Can be used for pedicled vascularized grafts from the distal radius (1–2 intercompartmental supraretinacular artery); Better exposure for radial styloidectomy in the case of radio-scaphoid arthritis	Disruption of the important dorsal vascular supply of the scaphoid
b) Single volar approach (Current study)	Single approach for scaphoid and graft donor area; Better correction of volar overhang and humpback deformity; Posterior vascularity and shell left intact; Better exposure for screw placement in small proximal pole fractures	

The single volar approach in our study was also amenable for both fixation and graft harvest, thereby reducing additional morbidity compared to other techniques (Table 3). Also, it was noted that in proximal pole fractures, screw placement was to be done perpendicular to the fracture line when possible, rather than along the long axis of the scaphoid to obtain better purchase in the small proximal fragment and minimize screw cut out, as the orientation of fracture is often oblique. Hence, the entry point of the screw had to be more volar, which was further aided by the volar approach. The distal radius metaphyseal donor site healed quite early with a mean period of 4 months except for two cases, which showed a persistent gap. Moreover, we believe that distal radius graft aids in foraging and increasing vascularity in the wrist, thereby contributing to healing.

The lack of functional evaluation and a control group are weaknesses of this study. Also, a computerized tomography scan (CT) would have been a definitive imaging modality for confirming union. However, being a retrospective study and serial X-rays being the imaging modality as per protocol, CT scans were not done. Besides, the retrospective nature of the study is also a reason for the unequal gender distribution as per presentation in the study period. The comparatively younger age of our study cohort and considerably early presentation may have a positive effect on the healing as proven earlier by studies [5].

## Conclusion

Though our small sample size does not permit a definitive conclusion, bony union achieved in all patients, maintenance of alignment parameters, minimal complications and a single volar approach for both correction of deformity and graft harvest, justifies our technique as a novel and reproducible one in the management of different types of scaphoid nonunion at any level.

## Data Availability

Not applicable.
